# Supplementary material for: Association between Sedentary Behavior and Cognitive Performance in Middle-Aged and Elderly Adults: Cross-Sectional Results from ELSA-Brasil
Source: Int J Environ Res Public Health. 2022 Oct 31;19(21):14234. doi: 10.3390/ijerph192114234 (PMC9654160; doi:10.3390/ijerph192114234)
Supplement: Supplementary file 1 [file ijerph-19-14234-s001.zip › ijerph-1943436-supplementary.pdf]

Table S1. Score on cognitive function tests according to sociodemographic characteristics and physical activity of the sample stratified by sex. ELSA-Brasil (2012-2014)

	**Total**		**Men**		**Women**	
	**Cognitive performance**		**Cognitive performance**		**Cognitive performance**	
	**Mean (SD)**	**IC 95%**	**Mean (SD)**	**IC 95%**	**Mean (SD)**	**IC 95%**
**Memory***						
Schooling	**n=6505**		**n=2915**		**n=3590**	
Incomplete elementary school	32.50 (6.35)	31.84 - 33.17	31.44 (6.23)	30.60 - 32.28	34.14 (6.21)	33.10 - 35.18
Complete elementary school	33.60 (6.10)	33.08 - 34.13	32.65 (6.00)	31.92 - 33.37	34.60 (6.05)	33.85 - 35.35
High school	36.15 (5.84)	35.88 - 36.42	34.51 (5.82)	34.09 - 34.92	37.29 (5.58)	36.96 - 37.63
Higher education	39.07 (5.61)	38.89 - 39.25	37.61 (5.73)	37.34 - 37.89	40.23 (5.24)	40.01 - 40.45
Race/skin colour, n (%)	**n=6415**		**n=2866**		**n=3549**	
Black	35.85 (5.90)	35.47 - 36.23	34.04 (5.91)	33.41 - 34.66	36.93 (5.63)	36.47 - 37.39
Brown	36.65 (6.23)	36.35 - 36.95	34.84 (6.37)	34.38 - 35.31	38.14 (5.69)	37.77 - 38.51
White	38.29 (6.02)	38.09 - 38.49	36.85 (5.99)	36.57 - 37.14	39.56 (5.75)	39.30 - 39.82
Yellow	37.16 (5.93)	36.32 - 38.01	33.91 (5.95)	32.48 - 35.34	38.99 (5.10)	38.07 - 39.90
Indigenous	34.02 (6.83)	32.39 - 35.65	32.09 (6.42)	29.74 - 34.45	35.56 (6.85)	33.34 - 37.78
Socioeconomic position, n (%)	**n=6381**		**n=2868**		**n=3513**	
High	39.01 (5.67)	38.79 - 39.23	37.57 (5.74)	37.26 - 37.87	40.52 (5.20)	40.23 - 40.81
Medium	37.36 (6.02)	37.11 - 37.61	35.58 (6.07)	35.15 - 36.00	38.33 (5.76)	38.03 - 38.62
Low	34.76 (6.22)	34.45 - 35.08	33.08 (6.08)	32.64 - 33.52	36.39 (5.91)	35.97 - 36.81

Marital status, n (%)	**n=6505**		**n=2915**		**n=3590**	
Married/stable union	37.20 (6.23)	37.01 - 37.40	35.92 (6.21)	35.67 - 36.17	39.15 (5.76)	38.86 - 39.44
Divorced	37.91 (5.98)	37.57 - 38.26	35.76 (5.97)	35.10 - 36.41	38.74 (5.78)	38.35 - 39.13
Widower	36.53 (6.15)	36.00 - 37.07	33.78 (6.33)	32.23 - 35.34	36.94 (6.03)	36.38 - 37.50
Single	38.42 (5.71)	38.04 - 38.81	36.57 (6.20)	35.54 - 37.59	38.79 (5.54)	38.38 - 39.20
Other	39.07 (6.20)	37.11 - 41.03	39.06 (6.73)	35.47 - 42.65	39.08 (5.98)	36.60 - 41.55
Physical Activity						
Inactive	37.00 (6.21)	36.78 - 37.21	35.51 (6.28)	35.18 - 35.84	38.09 (5.92)	37.82 - 38.36
Active	37.90 (6.04)	37.69 - 38.10	36.25 (6.10)	35.94 - 36.55	39.37 (5.58)	39.11 - 39.64
**Language****						
Schooling	**n=6505**		**n=2915**		**n=3590**	
Incomplete elementary school	19.43 (6.05)	18.80 - 20.07	18.83 (6.12)	18.00 - 19.65	20.37 (5.84)	19.39 - 21.34
Complete elementary school	21.83 (6.30)	21.28 - 22.37	21.15 (6.32)	20.38 - 21.91	22.53 (6.22)	21.77 - 23.30
High school	25.45 (7.09)	25.13 - 25.78	23.96 (6.94)	23.47 - 24.46	26.50 (7.02)	26.08 - 26.91
Higher education	31.36 (7.61)	31.11 - 31.60	30.29 (7.51)	29.93 - 30.65	32.20 (7.58)	31.88 - 32.53
Race/skin colour, n (%)	**n=6415**		**n=2866**		**n=3549**	
Black	25.35 (7.78)	24.85 - 25.85	23.63 (7.26)	22.86 - 24.40	26.37 (7.90)	25.73 - 27.02
Brown	26.36 (8.03)	25.96 - 26.75	24.54 (7.82)	23.97 - 25.10	27.85 (7.90)	27.33 - 28.37
White	29.94 (8.07)	29.67 - 30.20	28.76 (8.04)	28.37 - 29.14	30.98 (7.95)	30.62 - 31.33
Yellow	28.58 (8.27)	27.40 - 29.76	25.50 (8.71)	23.41 - 27.60	30.30 (7.52)	28.96 - 31.65
Indigenous	23 (7.58)	21.19 - 24.80	22.19 (7.90)	19.29 - 25.09	23.64 (7.36)	21.25 - 26.02

Socioeconomic position, n (%)	**n=6381**		**n=2868**		**n=3513**	
High	31.68 (7.63)	31.39 - 31.97	30.51 (7.48)	30.11 - 30.91	32.9 (7.60)	32.48 - 33.31
Medium	27.44 (7.82)	27.12 - 27.77	25.79 (7.85)	25.24 - 26.34	28.34 (7.66)	27.95 - 28.73
Low	23.49 (7.35)	23.12 - 23.86	21.79 (6.83)	21.30 - 22.28	25.13 (7.46)	24.61 - 25.66
Marital status, n (%)	**n=6505**		**n=2915**		**n=3590**	
Married/stable union	28.00 (8.32)	27.74 - 28.26	26.95 (8.23)	26.62 - 27.28	29.60 (8.20)	29.19 - 30.01
Divorced	28.87 (8.15)	28.40 - 29.35	26.88 (8.04)	26.00 - 27.77	29.64 (8.07)	29.09 - 30.18
Widower	27.41 (8.39)	26.69 - 28.14	24.22 (8.22)	22.20 - 26.25	27.88 (8.32)	27.11 - 28.65
Single	29.01 (8.01)	28.47 - 29.55	28.45 (8.84)	26.98 - 29.91	29.12 (7.84)	28.55 - 29.70
Other	32.53 (8.42)	29.87 - 35.19	30.31 (6.43)	26.88 - 33.74	33.96 (9.32)	30.11 - 37.80
Physical Activity						
Inactive	27.32 (8.18)	27.03 - 27.60	26.13 (8.20)	25.70 - 26.57	28.19 (8.06)	27.82 - 28.55
Active	29.22 (8.26)	28.94 - 29.50	27.72 (8.22)	27.31 - 28.13	30.57 (8.06)	30.19 - 30.95
**Executive function*****						
Schooling	**n=6505**		**n=2915**		**n=3590**	
Incomplete elementary school	271.18 (192.08)	251.13 - 291.23	275.89 (203.55)	248.52 - 303.25	263.95 (173.47)	234.96 - 292.94
Complete elementary school	205.88 (112.61)	196.15 - 215.61	208.96 (115.95)	194.88 - 223.04	202.69 (109.18)	189.20 - 216.18
High school	163.80 (91.61)	159.62 - 167.99	169.16 (90.92)	162.68 - 175.64	160.06 (91.94)	154.59 - 165.53
Higher education	102.01 (50.45)	100.40 - 103.61	102.40 (55.95)	99.72 - 105.07	101.70 (45.62)	99.75 - 103.64
Race/skin colour, n (%)	**n=6415**		**n=2866**		**n=3549**	
Black	181.27 (120.67)	173.47 - 189.06	193.70 (135.69)	179.31 - 208.09	173.88 (110.25)	164.88 - 182.88
Brown	158.18 (102.79)	153.19 - 163.17	165.55 (105.13)	157.95 - 173.15	152.12 (100.48)	145.53 - 158.71

White	115.38 (72.16)	113.02 - 117.74	119.97 (81.99)	116.05 - 123.89	111.33 (61.96)	108.55 - 114.11
Yellow	123.34 (81.01)	111.81 - 134.87	133.14 (84.39)	112.87 - 153.41	117.84 (78.87)	103.76 - 131.92
Indigenous	215.2 (175.88)	173.26 - 257.13	271.51 (226.71)	188.35 - 354.67	170.43 (104.64)	136.51 - 204.35
Socioeconomic position, n (%)	**n=6381**		**n=2868**		**n=3513**	
High	100.37 (51.88)	98.38 - 102.36	100.14 (52.71)	97.31 - 102.97	100.60 (51.02)	97.80 - 103.40
Medium	144.48 (87.45)	140.87 - 148.09	150.92 (90.73)	144.60 - 157.25	140.99 (85.45)	136.61 - 145.37
Low	191.81 (132.29)	185.13 - 198.49	210.53 (145.17)	200.06 - 220.99	173.78 (115.84)	165.58 - 181.98
Marital status, n (%)	**n=6505**		**n=2915**		**n=3590**	
Married/stable union	136.14 (97.76)	133.08 - 139.19	142.42 (107.73)	138.08 - 146.76	126.61 (79.41)	122.68 - 130.55
Divorced	130.22 (87.90)	125.15 - 135.29	138.30 (88.69)	128.55 - 148.06	127.13 (87.46)	121.20 - 133.06
Widower	161.02 (102.25)	152.17 - 169.88	162.98 (95.73)	139.45 - 186.51	160.74 (103.27)	151.16 - 170.32
Single	137.21 (90.95)	131.11 - 143.30	142.60 (100.62)	125.91 - 159.29	136.13 (88.94)	129.60 - 142.66
Other	109.92 (64.23)	89.65 - 130.20	96.56 (39.68)	75.41 - 117.70	118.48 (75.48)	87.32 - 149.63
Physical Activity	**n=6505**		**n=2915**		**n=3590**	
Inactive	145.41 (103.61)	141.84 - 148.98	150.22 (114.00)	144.18 - 156.26	141.89 (95.14)	137.57 - 146.20
Active	128.72 (86.31)	125.76 - 131.68	135.06 (95.79)	130.28 - 139.84	123.03 (76.40)	119.42 - 126.64

SD: standard deviation; 95% CI: 95% Confidence Interval. * Learning, recall and word recognition: number of correct words: score ranges from 0 to 50. ** Semantic and phonemic verbal fluency: number of words remembered in 1 minute. *** Trail test (part B): time (in seconds) to perform the test.

Table S2. Sitting time (in hours/day) during the week and weekend according to sociodemographic characteristics and physical activity in the sample stratified by sex. ELSA-Brasil (2012-2014)

Sedentary behaviour	Total		Men		Women	
	**Mean (SD)**	**IC 95%**	**Mean (SD)**	**IC 95%**	**Mean (SD)**	**IC 95%**
**Sitting time (hours/day) - Weekdays**						
Schooling	**n=6505**		**n=2915**		**n=3590**	
Incomplete elementary school	3.40 (2.88)	3.10 - 3.70	3.72 (2.91)	3.32 - 4.11	2.92 (2.78)	2.45 - 3.38
Complete elementary school	3.88 (2.89)	3.63 - 4.13	4.15 (2.87)	3.80 - 4.49	3.60 (2.88)	3.25 - 3.96
High school	4.40 (3.01)	4.26 - 4.54	4.72 (3.12)	4.49 - 4.94	4.18 (2.91)	4.02 - 4.36
Higher education	6.53 (3.14)	6.43 - 6.63	6.89 (3.07)	6.74 - 7.04	6.23 (3.17)	6.10 - 6.37
Race/skin colour, n (%)	**n=6415**		**n=2866**		**n=3549**	
Black	4.32 (2.93)	4.13 - 4.51	4.71 (3.00)	4.40 - 5.03	4.08 (2.86)	3.85 - 4.31
Brown	4.91 (3.35)	4.75 - 5.08	5.01 (3.22)	4.78 - 5.25	4.83 (3.45)	4.60 - 5.06
White	6.12 (3.19)	6.01 - 6.22	6.41 (3.22)	6.25 - 6.56	5.86 (3.14)	5.72 - 6.00
Yellow	6.17 (3.53)	5.67 - 6.67	6.60 (3.64)	5.72 - 7.47	5.93 (3.45)	5.31 - 6.55
Indigenous	3.89 (2.48)	3.30 - 4.48	3.86 (2.97)	2.76 - 4.95	3.92 (2.05)	3.25 - 4.59
Socioeconomic position, n (%)	**n=6381**		**n=2868**		**n=3513**	
High	6.67 (3.16)	6.54 - 6.79	7.00 (3.07)	6.83 - 7.16	6.32 (3.22)	6.14 - 6.50
Medium	5.17 (3.23)	5.04 - 5.31	5.48 (3.23)	5.26 - 5.71	5.01 (3.21)	4.84 - 5.17
Low	4.12 (2.91)	3.97 - 4.27	4.11 (2.86)	3.91 - 4.32	4.12 (2.95)	3.91 - 4.33

Marital status, n (%)	**n=6505**		**n=2915**		**n=3590**	
Married/stable union	5.57 (3.24)	5.47 - 5.67	5.79 (3.28)	5.66 - 5.92	5.25 (3.15)	5.09 - 5.40
Divorced	5.78 (3.40)	5.58 - 5.98	6.22 (3.37)	5.85 - 6.59	5.61 (3.40)	5.38 - 5.84
Widower	4.56 (3.09)	4.29 - 4.83	4.76 (3.10)	4.00 - 5.52	4.53 (3.09)	4.24 - 4.82
Single	5.56 (3.36)	5.34 - 5.79	6.17 (3.33)	5.62 - 6.73	5.44 (3.36)	5.19 - 5.69
Other	7.09 (2.76)	6.22 - 7.96	7.18 (2.07)	6.08 - 8.29	7.04 (3.16)	5.73 - 8.34
Physical Activity	**n=6505**		**n=2915**		**n=3590**	
Inactive	5.52 (3.35)	5.40 - 5.63	5.92 (3.39)	5.74 - 6.10	5.22 (3.29)	5.07 - 5.37
Active	5.56 (3.22)	5.45 - 5.67	5.77 (3.20)	5.61 - 5.93	5.37 (3.23)	5.22 - 5.52
**Sitting time (hours/day) - Weekends**						
Schooling	**n=6505**		**n=2915**		**n=3590**	
Incomplete elementary school	3.29 (2.53)	3.02 - 3.55	3.67 (2.73)	3.31 - 4.04	2.70 (2.08)	2.35 - 3.04
Complete elementary school	3.69 (2.77)	3.45 - 3.93	3.84 (2.83)	3.50 - 4.19	3.53 (2.71)	3.19 - 3.86
High school	3.74 (2.59)	3.62 - 3.86	4.16 (2.76)	3.96 - 4.35	3.45 (2.42)	3.31 - 3.59
Higher education	5.21 (2.94)	5.12 - 5.31	5.66 (3.08)	5.51 - 5.81	4.85 (2.78)	4.73 - 4.97
Race/skin colour, n (%)	**n=6415**		**n=2866**		**n=3549**	
Black	3.76 (2.82)	3.58 - 3.94	4.28 (3.24)	3.94 - 4.63	3.45 (2.50)	3.25 - 3.66
Brown	4.08 (2.80)	3.95 - 4.22	4.28 (2.82)	4.07 - 4.48	3.93 (2.78)	3.74 - 4.11
White	4.99 (2.90)	4.89 - 5.08	5.36 (3.03)	5.22 - 5.51	4.65 (2.74)	4.53 - 4.77
Yellow	4.74 (2.92)	4.33 - 5.16	5.29 (3.28)	4.50 - 6.08	4.43 (2.65)	3.96 - 4.91

Indigenous	3.66 (2.75)	3.00 - 4.32	4.05 (3.45)	2.78 - 5.32	3.36 (2.04)	2.69 - 4.02
Socioeconomic position, n (%)	**n=6381**		**n=2868**		**n=3513**	
High	5.38 (2.95)	5.26 - 5.49	5.80 (3.03)	5.64 - 5.97	4.93 (2.80)	4.78 - 5.08
Medium	4.20 (2.80)	4.08 - 4.31	4.55 (2.98)	4.35 - 4.76	4.00 (2.68)	3.87 - 4.14
Low	3.70 (2.64)	3.57 - 3.83	3.88 (2.74)	3.68 - 4.08	3.53 (2.53)	3.35 - 3.71
Marital status, n (%)	**n=6505**		**n=2915**		**n=3590**	
Married/stable union	4.60 (2.91)	4.51 - 4.69	4.92 (3.05)	4.80 - 5.04	4.12 (2.60)	3.99 - 4.25
Divorced	4.59 (2.96)	4.42 - 4.76	5.20 (3.16)	4.85 - 5.54	4.36 (2.85)	4.17 - 4.55
Widower	3.94 (2.58)	3.72 - 4.16	4.11 (2.79)	3.42 - 4.80	3.91 (2.55)	3.68 - 4.15
Single	4.72 (3.02)	4.52 - 4.92	5.36 (3.08)	4.85 - 5.87	4.59 (2.99)	4.37 - 4.81
Other	5.43 (2.63)	4.60 - 6.27	5.81 (2.63)	4.40 - 7.21	5.20 (2.66)	4.09 - 6.3
Physical Activity	**n=6505**		**n=2915**		**n=3590**	
Inactive	4.58 (3.05)	4.47 - 4.68	5.13 (3.23)	4.96 - 5.30	4.17 (2.84)	4.04 - 4.30
Active	4.56 (2.77)	4.47 - 4.66	4.80 (2.90)	4.66 - 4.95	4.34 (2.63)	4.22 - 4.47

SD: standard deviation; 95% CI: 95% Confidence Interval.

Tabela S3. Leisure and occupational screen time (in hours/day) during the week and weekend according to sociodemographic characteristics and physical activity of the sample stratified by sex. ELSA-Brasil (2012-2014)

Sedentary behaviour	Total		Men		Women	
	**Mean (SD)**	**IC 95%**	**Mean (SD)**	**IC 95%**	**Mean (SD)**	**IC 95%**
**Leisure Screen time (hours/day) - Weekdays**						
Schooling	**n=6505**		**n=2915**		**n=3590**	
Incomplete elementary school	2.40 (2.02)	2.19 - 2.61	2.59 (2.19)	2.30 - 2.89	2.10 (1.68)	1.82 - 2.38
Complete elementary school	2.80 (2.11)	2.62 - 2.98	2.75 (1.88)	2.52 - 2.98	2.85 (2.33)	2.56 - 3.14
High school	2.49 (1.92)	2.41 - 2.58	2.67 (2.01)	2.52 - 2.81	2.37 (1.84)	2.26 - 2.48
Higher education	2.36 (1.56)	2.31 - 2.41	2.32 (1.50)	2.25 - 2.39	2.40 (1.60)	2.33 - 2.46
Race/skin colour, n (%)	**n=6415**		**n=2866**		**n=3549**	
Black	2.49 (2.07)	2.35 - 2.62	2.74 (2.35)	2.49 - 2.99	2.34 (1.87)	2.18 - 2.49
Brown	2.43 (1.78)	2.34 - 2.51	2.50 (1.73)	2.38 - 2.63	2.36 (1.81)	2.24 - 2.48
White	2.44 (1.65)	2.38 - 2.49	2.41 (1.62)	2.34 - 2.49	2.45 (1.67)	2.38 - 2.53
Yellow	2.39 (1.74)	2.14 - 2.63	2.30 (1.47)	1.94 - 2.65	2.43 (1.88)	2.10 - 2.77
Indigenous	2.40 (1.56)	2.03 - 2.78	2.37 (1.32)	1.88 - 2.85	2.44 (1.74)	1.87 - 3.00
Socioeconomic position, n (%)	**n=6381**		**n=2868**		**n=3513**	
High	2.30 (1.58)	2.24 - 2.36	2.30 (1.61)	2.22 - 2.39	2.29 (1.55)	2.20 - 2.38
Medium	2.51 (1.79)	2.44 - 2.58	2.68 (1.79)	2.55 - 2.80	2.42 (1.78)	2.33 - 2.51
Low	2.56 (1.94)	2.46 - 2.66	2.55 (1.90)	2.42 - 2.69	2.57 (1.97)	2.43 - 2.71
Marital status, n (%)	**n=6505**		**n=2915**		**n=3590**	
Married/stable union	2.33 (1.66)	2.28 - 2.39	2.44 (1.73)	2.37 - 2.51	2.17 (1.52)	2.09 - 2.24

Divorced	2.55 (1.90)	2.44 - 2.66	2.62 (1.76)	2.43 - 2.82	2.52 (1.95)	2.39 - 2.65
Widower	2.54 (1.76)	2.38 - 2.69	2.62 (1.75)	2.18 - 3.05	2.52 (1.76)	2.36 - 2.69
Single	2.69 (1.89)	2.57 - 2.82	2.52 (1.93)	2.18 - 3.05	2.73 (1.88)	2.59 - 2.87
Other	2.27 (1.33)	1.85 - 2.69	1.98 (0.98)	1.46 - 2.51	2.46 (1.50)	1.83 - 3.08
Physical Activity	**n=6505**		**n=2915**		**n=3590**	
Inactive	2.52 (1.89)	2.46 - 2.59	2.63 (1.96)	2.53 - 2.74	2.44 (1.84)	2.36 - 2.52
Active	2.35 (1.58)	2.30 - 2.40	2.32 (1.51)	2.25 - 2.40	2.37 (1.64)	2.30 - 2.45
**Leisure Screen time (hours/day) - Weekends**						
Schooling	**n=6505**		**n=2915**		**n=3590**	
Incomplete elementary school	2.81 (2.37)	2.56 - 3.06	3.01 (2.38)	2.69 - 3.33	2.51 (2.33)	2.12 - 2.90
Complete elementary school	3.13 (2.47)	2.92 - 3.35	3.21 (2.16)	2.95 - 3.47	3.05 (2.76)	2.71 - 3.40
High school	2.99 (2.24)	2.88 - 3.09	3.22 (2.29)	3.05 - 3.38	2.82 (2.19)	2.69 - 2.95
Higher education	2.96 (1.89)	2.90 - 3.02	3.05 (1.92)	2.95 - 3.14	2.89 (1.87)	2.81 - 2.97
Race/skin colour, n (%)	**n=6415**		**n=2866**		**n=3549**	
Black	2.89 (2.32)	2.74 - 3.04	3.13 (2.46)	2.86 - 3.39	2.75 (2.22)	2.57 - 2.93
Brown	2.92 (2.20)	2.81 - 3.03	3.11 (2.18)	2.95 - 3.26	2.76 (2.20)	2.62 - 2.91
White	3.04 (1.96)	2.97 - 3.10	3.12 (1.96)	3.03 - 3.21	2.96 (1.95)	2.88 - 3.05
Yellow	2.78 (2.07)	2.49 - 3.08	2.71 (1.75)	2.29 - 3.14	2.82 (2.24)	2.42 - 3.22
Indigenous	2.49 (1.94)	2.03 - 2.96	2.41 (1.99)	1.68 - 3.14	2.56 (1.92)	1.94 - 3.18
Socioeconomic position, n (%)	**n=6381**		**n=2868**		**n=3513**	
High	2.88 (1.84)	2.81 - 2.95	3.02 (1.90)	2.92 - 3.12	2.73 (1.76)	2.63 - 2.83
Medium	3.07 (2.19)	2.98 - 3.16	3.35 (2.22)	3.19 - 3.50	2.93 (2.16)	2.81 - 3.04

Low	2.97 (2.27)	2.86 - 3.09	2.99 (2.19)	2.83 - 3.14	2.96 (2.35)	2.79 - 3.13
Marital status, n (%)	**n=6505**		**n=2915**		**n=3590**	
Married/stable union	2.94 (2.00)	2.88 - 3.00	3.11 (2.09)	3.03 - 3.20	2.67 (1.84)	2.58 - 2.76
Divorced	2.96 (2.12)	2.84 - 3.08	3.11 (2.07)	2.89 - 3.34	2.90 (2.14)	2.75 - 3.04
Widower	2.84 (2.09)	2.66 - 3.02	2.86 (1.90)	2.39 - 3.32	2.84 (2.12)	2.64 - 3.03
Single	3.23 (2.31)	3.08 - 3.39	2.98 (2.07)	2.64 - 3.32	3.28 (2.36)	3.11 - 3.46
Other	2.79 (1.68)	2.25 - 3.32	3.15 (2.12)	2.01 - 4.28	2.56 (1.33)	2.00 - 3.11
Physical Activity	**n=6505**		**n=2915**		**n=3590**	
Inactive	3.12 (2.27)	3.04 - 3.20	3.33 (2.28)	3.21 - 3.45	2.97 (2.25)	2.87 - 3.07
Active	2.82 (1.85)	2.76 - 2.88	2.90 (1.86)	2.81 - 2.99	2.75 (1.84)	2.66 - 2.84
**Occupational screen time (hours/day) - Weekdays**						
Schooling	**n=6505**		**n=2915**		**n=3590**	
Incomplete elementary school	0.71 (2.01)	0.50 - 0.92	0.87 (2.06)	0.59 - 1.14	0.48 (1.92)	0.16 - 0.80
Complete elementary school	1.06 (2.01)	0.89 - 1.24	1.24 (2.06)	0.99 - 1.49	0.88 (1.94)	0.64 - 1.12
High school	2.15 (2.79)	2.03 - 2.28	2.36 (2.79)	2.17 - 2.56	2.01 (2.79)	1.84 - 2.17
Higher education	4.12 (3.03)	4.03 - 4.22	4.38 (2.80)	4.25 - 4.51	3.92 (3.19)	3.78 - 4.05
Race/skin colour, n (%)	**n=6415**		**n=2866**		**n=3549**	
Black	2.05 (2.67)	1.88 - 2.22	2.18 (2.71)	1.89 - 2.46	1.97 (2.64)	1.76 - 2.19
Brown	2.56 (2.96)	2.41 - 2.70	2.59 (2.88)	2.38 - 2.80	2.53 (3.02)	2.33 - 2.72
White	3.64 (3.13)	3.53 - 3.74	3.81 ()2.96	3.67 - 3.96	3.48 (3.27)	3.33 - 3.63
Yellow	3.85 (3.41)	3.37 - 4.34	4.11 (3.18)	3.34 - 4.87	3.71 (3.53)	3.08 - 4.34

Indigenous	1.81 (2.49)	1.22 - 2.41	2.05 (2.71)	1.06 - 3.05	1.62 (2.31)	0.87 - 2.37
Socioeconomic position, n (%)	**n=6381**		**n=2868**		**n=3513**	
High	4.14 (2.97)	4.02 - 4.25	4.41 (2.80)	4.26 - 4.56	3.86 (3.11)	3.69 - 4.03
Medium	3.04 (3.13)	2.91 - 3.17	3.33 (3.04)	3.11 - 3.54	2.88 (3.17)	2.72 - 3.04
Low	1.54 (2.54)	1.41 - 1.67	1.32 (2.15)	1.16 - 1.48	1.76 (2.84)	1.56 - 1.96
Marital status, n (%)	**n=6505**		**n=2915**		**n=3590**	
Married/stable union	3.22 (3.06)	3.13 - 3.32	3.30 (3.00)	3.18 - 3.42	3.10 (3.15)	2.95 - 3.26
Divorced	3.35 (3.18)	3.16 - 3.53	3.43 (2.97)	3.10 - 3.75	3.32 (3.26)	3.09 - 3.54
Widower	1.96 (2.63)	1.73 - 2.19	2.43 (2.94)	1.71 - 3.15	1.89 (2.58)	1.65 - 2.13
Single	3.07 (3.22)	2.86 - 3.29	3.59 (2.81)	3.12 - 4.05	2.97 (3.29)	2.73 - 3.21
Other	4.68 (3.17)	3.68 - 5.68	4.04 (2.72)	2.59 - 5.49	5.09 (3.42)	3.67 - 6.50
Physical Activity						
Inactive	3.06 (3.17)	2.95 - 3.17	3.20 (3.08)	3.04 - 3.36	2.96 (3.23)	2.81 - 3.11
Active	3.20 (3.02)	3.10 - 3.31	3.41 (2.90)	3.27 - 3.56	3.02 (3.12)	2.87 - 3.17
**Occupational screen time (hours/day) - Weekends**						
Schooling	**n=6505**		**n=2915**		**n=3590**	
Incomplete elementary school	0.45 (1.59)	0.28 - 0.61	0.64 (1.93)	0.38 - 0.90	0.14 (0.70)	0.02 - 0.26
Complete elementary school	0.47 (1.25)	0.36 - 0.58	0.61 (1.37)	0.44 - 0.78	0.32 (1.09)	0.19 - 0.46
High school	0.75 (1.54)	0.67 - 0.82	0.98 (1.79)	0.85 - 1.11	0.58 (1.32)	0.50 - 0.66
Higher education	1.74 (2.03)	1.67 - 1.80	1.94 (2.01)	1.85 - 2.04	1.57 (2.03)	1.49 - 1.66

Race/skin colour, n (%)	**n=6415**		**n=2866**		**n=3549**	
Black	0.77 (1.49)	0.68 - 0.87	0.95 (1.76)	0.76 - 1.13	0.67 (1.30)	0.56 - 0.77
Brown	1.08 (1.78)	0.99 - 1.16	1.23 (1.97)	1.09 - 1.38	0.95 (1.60)	0.84 - 1.05
White	1.49 (2.01)	1.42 - 1.55	1.67 (1.99)	1.57 - 1.76	1.32 (2.01)	1.23 - 1.41
Yellow	1.41 (1.83)	1.15 - 1.67	1.50 (1.92)	1.04 - 1.96	1.36 (1.79)	1.04 - 1.68
Indigenous	0.78 (1.36)	0.46 - 1.11	0.74 (1.16)	0.31 - 1.16	0.82 (1.51)	0.32 - 1.31
Socioeconomic position, n (%)	**n=6381**		**n=2868**		**n=3513**	
High	1.96 (2.11)	1.88 - 2.04	2.07 (2.03)	1.96 - 2.18	1.84 (2.19)	1.72 - 1.96
Medium	0.95 (1.63)	0.88 - 1.02	1.12 (1.82)	1.00 - 1.25	0.86 (1.51)	0.78 - 0.93
Low	0.61 (1.51)	0.53 - 0.69	0.78 (1.73)	0.66 - 0.91	0.44 (1.23)	0.36 - 0.53
Marital status, n (%)	**n=6505**		**n=2915**		**n=3590**	
Married/stable union	1.35 (1.92)	1.29 - 1.41	1.48 (1.97)	1.40 - 1.56	1.16 (1.83)	1.07 - 1.25
Divorced	1.31 (1.90)	1.20 - 1.42	1.50 (2.08)	1.27 - 1.73	1.24 (1.82)	1.11 - 1.36
Widower	0.72 (1.34)	0.60 - 0.83	1.10 (1.75)	0.67 - 1.53	0.66 (1.27)	0.54 - 0.78
Single	1.26 (2.06)	1.12 - 1.40	1.63 (2.00)	1.30 - 1.97	1.19 (2.07)	1.04 - 1.34
Other	1.85 (1.97)	1.23 - 2.47	1.09 (1.11)	0.50 - 1.68	2.34 (2.25)	1.40 - 3.27
Physical Activity						
	**n=6505**		**n=2915**		**n=3590**	
Inactive	1.22 (1.97)	1.15 - 1.29	1.45 (2.12)	1.34 - 1.57	1.05 (1.83)	0.96 - 1.13
Active	1.35 (1.84)	1.29 - 1.41	1.50 (1.85)	1.40 - 1.59	1.22 (1.83)	1.13 - 1.30

SD: standard deviation; 95% CI: 95% Confidence Interval.
